# A qualitative exploration of Australian parental perspectives of commercial squeeze pouches for infants and children

**DOI:** 10.1093/heapro/daaf212

**Published:** 2025-12-10

**Authors:** Bianca Smith, Catharine A K Fleming, Ami Seivwright, Yasmin Mistry, Katherine Kent

**Affiliations:** School of Medical, Indigenous and Health Sciences, University of Wollongong, Wollongong, NSW 2522, Australia; School of Health Sciences, Western Sydney University, Sydney, NSW 2560, Australia; Monash Climate Change Communication Research Hub, Monash University, Melbourne, VIC 3000, Australia; Department of Natural Sciences, Design and Sustainable Development, Mid Sweden University, Akademigatan 1, SE-831 25 Ostersund, Sweden; School of Medical, Indigenous and Health Sciences, University of Wollongong, Wollongong, NSW 2522, Australia; School of Medical, Indigenous and Health Sciences, University of Wollongong, Wollongong, NSW 2522, Australia

**Keywords:** commercial complementary foods, infant squeeze pouch, infant nutrition, consumer behaviour, parental feeding perspectives

## Abstract

Foods in commercial squeeze pouches are a widely used feeding option for children, yet growing concerns exist about their nutritional quality and impacts on feeding development. There is limited evidence on how and why parents use these products, leaving critical gaps in understanding how to design policy and food environments that support positive nutrition choices. This study aimed to explore Australian parents’ experiences and motivations for providing commercial squeeze pouches to infants and children. An online survey was distributed nationally to parents, containing an open-ended question inviting them to describe the role of squeeze pouches in their child’s diet and their reasons for use. Responses were inductively thematically analysed to understand motivations, perceptions, experiences, and feeding contexts. A total of 179 parents, mainly mothers (78.1%), participated, revealing five intersecting themes: *societal and behavioural drivers*, *feeding supplement strategies*, *feeding confidence and nutritional perceptions*, *commercial food environment*, and *environmental impact considerations* that influenced their use of squeeze pouches when feeding their child. Within the themes, parents frequently described how squeeze pouches are a practical solution to managing time pressures, fussy or neurodivergent eating behaviours, and feeding during illness or travel. However, many also expressed concerns about cost, packaging waste, and feeding skill development. Overall, a broader societal paradox emerged where convenience and modern parenting demands often outweighed nutritional or environmental ideals. The complex insights provided by parents demonstrate the need for policy and practice responses that address structural and commercial drivers of food choice, while supporting families with accessible, evidence-based feeding guidance.

Contribution to Health Promotion

- This research explores and provides novel insights into Australian parents’ motivations, experiences, and reasons for providing commercial squeeze pouches to their children (0–17 years).
- Highlights that parents’ decisions to use commercial squeeze pouches are influenced by a combination of both societal and commercial determinants of health.
- These insights highlight the need for targeted public health promotion messages and policies that address the nutritional composition and promotional marketing of these products to support healthier feeding practices among Australian families that align with national dietary guidelines.

## INTRODUCTION

The proliferation of commercial baby and toddler foods, including squeeze pouches, reflects a broader shift in the global food system towards the commodification of infant and child feeding (Bentley 2014, World Health Organization 2019, Garcia *et al*. 2020, Shyam *et al*. 2025). In recent years, the Australian infant and child feeding landscape has been shaped by the rapid growth of commercially produced packaged foods, particularly foods in squeeze pouches, pureed or smooth textured foods marketed in squeezable plastic containers with spouts, which now dominate the Australian baby food market, contributing to sales worth 1.2 billion (AUD) (Brunacci *et al*. 2023). These products are positioned as convenient, mess-free, and healthy options for young children and for time-poor parents (Dunford *et al*. 2024). While originally designed for complementary feeding in infancy, squeeze pouches are now widely consumed across the early years (Haszard *et al*. 2024, McLean *et al*. 2024), including among toddlers and young school-aged children among high-income countries (Smith *et al*. 2025). A recent Australian study has shown that infants and younger children (ages 0–5 years) consume these products frequently (weekly consumption), while older children and even adolescents (ages 6–17 years) consumed dairy-based squeeze pouches, though less often (Kent *et al*. 2025). Shifts in the food supply are occurring within an increasingly commercialized food environment, where marketing practices, pricing strategies, and on-pack health claims significantly shape consumer behaviour, including among parents making decisions about what and how to feed their children (Brunacci *et al*. 2023, Brand-Williamson *et al*. 2025).

Despite their popularity, these products raise important public health and developmental concerns. New research has revealed that none of the commercial infant and toddler foods in Australian supermarkets meet the WHO guidelines for product promotion, with squeeze pouches having the highest incidence of prohibited claims (Dunford *et al*. 2024). Australian and international studies have highlighted that squeeze pouches are often high in free sugars, low in iron (Brunacci *et al*. 2023). Repeated use beyond infancy, or repeated reliance on these products, may displace nutrient-rich, age-appropriate family foods and interfere with the development of key feeding skills (Kalhoff *et al*. 2024).

Parental feeding decisions are shaped by both the social and commercial determinants of health. The social determinants of health, such as income, time availability, employment status, education, and access to support systems, create the structural context in which parents make everyday food decisions (LaManna *et al*. 2024). In many households, these conditions present practical and emotional constraints, such as stress, decision fatigue, or concerns about providing adequate meals, making packaged products like foods in commercial squeeze pouches an attractive or necessary option for infant and child feeding. Alongside these structural conditions, the commercial determinants of health describe how corporate practices influence consumption patterns, policy environments, and health outcomes (Friel *et al*. 2023, Gilmore *et al*. 2023). In the context of infant and toddler feeding, the commercial determinants of health operate through targeted marketing, selective use of health and nutrition claims, and a regulatory environment that permits the promotion of ultra-processed foods to caregivers (Baker *et al*. 2021). These commercial influences may reinforce the normalization of squeeze pouch use and shape parental beliefs about what constitutes healthy or acceptable feeding (Rowan *et al*. 2022, Neve *et al*. 2024).

There are currently a limited number of qualitative studies from international settings, which show that parents often experience ambivalence when using squeeze pouches, valuing their practicality while expressing concern about their healthiness, cost, and impact on feeding development (Maslin *et al*. 2015, Isaacs *et al*. 2022, Spurlock *et al*. 2023). Yet in everyday contexts, structural constraints such as time scarcity, financial pressure, and limited institutional support can override ideals, contributing to a broader paradox in feeding decisions (Mohamad *et al*. 2019). In Australia, there is a critical gap in understanding how parents navigate these tensions in relation to commercial squeeze pouch use. Most existing studies focus on nutrient profiles (Brunacci *et al*. 2023, Dunford *et al*. 2024) or analyses of marketing claims (Brand-Williamson *et al*. 2025); thus, there remains a lack of research that has explored how Australian parents’ and caregivers’ motivations and experiences around the use of commercial squeeze pouch products with their children. This gap is particularly important in light of the recent directive by Australian food ministers to Food Standards Australia New Zealand (FSANZ) to review the labelling and composition of toddler and infant foods, including the potential regulation of marketing claims, as part of a broader agenda to strengthen the food regulatory system in support of public health (Food Regulation Standing Committee 2025). Qualitative evidence is critical to understanding the lived experiences, motivations, and trade-offs parents navigate when using squeeze pouches, which form the context for effective regulation and, more broadly, can inform the development of a healthy food environment. Therefore, the aim of this study was to explore parental experiences and motivations for providing commercial squeeze pouch products to infants and children in Australia, incorporating both perspectives of those who did or did not report use these products.

## METHODS

### Study design

The current study was part of a larger mixed methods study utilizing a cross-sectional survey of Australian parents to explore their children’s frequency, predictors, and patterns of commercial squeeze pouch consumption. The objective of the current paper was to further explore the complexities, experiences, and perceived benefits or concerns of commercial squeeze pouch use by parents. Utilizing an open-ended qualitative survey question within the cross-sectional survey provided an opportunity to gain a comprehensive, nuanced understanding of the complexities that drive parental feeding choices when using commercial squeeze pouches.

### Participants and recruitment

Adult parents and/or primary caregivers residing in Australia with children aged 0–17 years were eligible to participate. Recruitment occurred through multiple convenience sampling strategies. Participants were invited from an existing panel of Australian adults that had been recruited via social media, mainstream media (radio; newspapers), and a university website hosting a sign-up form, with surveys on social and health topics between 2020 and 2024. End of survey recruitment, where participants were asked to provide their details if they wanted to participate in further surveys, was also used to build the panel. Additional recruitment for this study utilized media and social media advertising. As this was an exploratory study, the sample size was not pre-determined.

### Data collection

Interested individuals were invited to access the self-administered survey via a Qualtrics link (Qualtrics, Provo, UT). Upon clicking the link, participants were presented with a participant information sheet and asked to confirm informed consent and eligibility (i.e. aged 18 years or older and residing in Australia) before beginning the survey. To ensure data quality and authenticity, survey data and metadata were examined to identify IP addresses, unusually short or long response times, identical survey responses, and straight-line responding (Malhotra 2008, Kim *et al*. 2019). Likely attributable to the lack of incentives for completion and the targeted nature of recruitment (e.g. posting to parent and community groups on Facebook versus large, general audiences), this examination did not identify any suspected false responses. Participants were asked a range of sociodemographic questions pertaining to gender (self-identified), age in years, whether they identify as Aboriginal and/or Torres Strait Islander, whether they have a disability (self-reported), postcode, highest level of education, income range, food insecurity, and household composition (i.e. how many children and their ages). The methods used to assess sociodemographic characteristics are described elsewhere (Kent *et al*. 2025). The survey consisted of a series of closed-ended questions about frequency of consumption of squeeze pouches and included an open-ended question designed to elicit qualitative parental reflections on squeeze pouch use which is the basis for the analysis presented in this manuscript: ‘Please describe the role that squeeze pouches and snacks play in your child's diet and daily routine’. While participants were asked about both snacks and squeeze pouch, only pouch-related data are reported in this study. Data collected between December 2023 and September 2024. Participants did not receive payment or incentives for their involvement in this study. Ethical approval was obtained from the University of Tasmania Human Research Ethics Committee (project ID: 20587).

### Data analysis

Open-ended survey responses were analysed using inductive thematic analysis to explore parental experiences and motivations or concerns of commercial squeeze pouch product use. The use of an inductive approach allowed for the development of themes to be data driven. The process involved all textual responses being exported from Qualtrics and analysed using Braun and Clarke’s six-phase framework for inductive thematic analysis (Braun and Clarke 2006). Initial familiarization occurred through repeated reading of the raw data. Two researchers (B.S., Y.S.) independently generated open codes using Microsoft Excel, identifying meaningful concepts and recurring patterns within the data. Coding was then then discussed collaboratively in detail with additional team members (C.A.K.F., K.K.) to reflect on interpretations and establish an agreed coding framework, resulting in the addition of a further two open codes. Through an iterative process of constant comparison, codes were grouped into subthemes and were used to identify broader higher-order themes that captured cross-cutting issues in parental reflections.

As nutrition-focused academics (K.K., C.A.K.F.) and students (B.S., Y.M.), our knowledge and experiences shape and inform our perspectives, interpretations, and the way we present our findings. In addition, two of the researchers (K.K. and C.A.K.F.) are parents with lived experience of the study focus (children aged within the target age range). Despite this, it is important to clarify that this does not imply an uncritical or biased portrayal of the realities being examined. Rather, we adopted a rigorous process of critical reflection to ensure that the narratives were grounded in the existing evidence base rather than opinion while also acknowledging that our nutrition-focused backgrounds and lived experiences with children of the target age range may have informed our interpretation of the data. To enhance rigour, we maintained an audit trail of coding decisions, documented researcher discussions, and held debriefing meetings to resolve discrepancies and ensure consistency. We prioritized analytical depth over frequency, focusing on the meaning and context of parental narratives.

## RESULTS

An overview of the sociodemographic characteristics of the survey sample is presented in Table 1. In total, there were 179 survey respondents who reported on squeeze pouch use in *n* = 248 infants and children. A total of 155 open-ended responses were available for analysis, ranging from single-word answers to responses of up to 89 words. This study forms part of a larger cross-sectional survey of 343 parents reporting on 493 children (Kent *et al*. 2025).

**Table 1.** Characteristics of study participants.

Demographic variable	*n*	%
Gender		
Male	39	21.9
Female	139	78.1
Age		
18–34 years	18	10.7
35–45 years	62	36.9
46+ years	88	52.4
Education level		
High school	10	5.60
TAFE^a^	37	20.9
University	130	73.4
Identify as Aboriginal and Torres Strait Islander		
Yes	6	3.40
No	170	96.6
Born in Australia		
No	28	15.8
Yes	149	84.2
Language other than English		
No	162	91.5
Yes	15	8.50
Household composition		
Couple with dependant child(ren)	140	80.0
Single parent with dependent	22	12.6
Multi-generational family	13	7.40
Employment status		
Employed	144	82.3
Not employed	31	17.7
Activity limiting health condition or disability		
No	119	67.6
Yes	57	32.4
Income		
$0–$1499	81	47.1
$1500–$2000	34	19.8
$2000+	57	33.1
Number of children in household, in each age category		
0–2 years	28	15.6
2–5 years	48	26.8
6–12 years	88	49.2
13–17 years	84	46.9
Food security status		
Food secure	102	57.0
Food insecure	77	43.0

^a^Technical and Further Education (TAFE) refers to government-funded institutes across Australia that deliver vocational education and training in a variety of occupational fields.

Most respondents were female (78%), and just over half of respondents were 46+ years of age (52%). A large proportion of survey respondents were born in Australia (84%), and only 9% of respondents reported speaking a language other than English. A small proportion of respondents identified as either Aboriginal and/or Torres Strait Islander (3%). The majority of respondents (68%) reported no activity limiting health condition or disability. Most respondents held a university degree (73%) (bachelor’s degree or higher), 82% of respondents were currently employed, and 47% reported household income of $0–$1500 per week. With regard to household composition, 80% of respondents were couples with dependent children. More than half of respondents experienced some degree of food security (57%).

**Figure 1.**
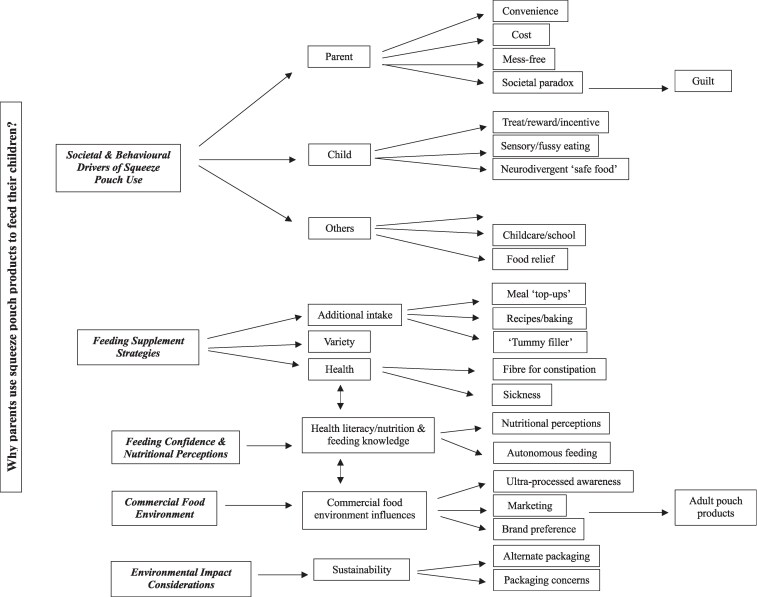
Thematic map illustrating the themes, sub-themes, and codes representing parental experiences with squeeze pouch use.

Five higher-order themes were developed from the inductive thematic analysis of parent responses: ‘(i) societal and behavioural drivers, (ii) feeding supplement strategies, (iii) feeding confidence and nutritional perceptions, (iv) commercial food environment, and (v) environmental impact considerations (Fig. 1)’. Subthemes are outlined in the thematic map (Fig. 1) and described in the analysis below.

The qualitative data analysis is presented below under five key themes: ‘societal and behavioural drivers, feeding supplement strategies, feeding confidence and nutritional perceptions, commercial food environment, and environmental impact considerations’. The themes capture the complex and multifaceted experiences of parents when using commercial squeeze pouch products to feed their child.

### Societal and behavioural drivers of squeeze pouch use

This theme describes the emotional, practical, and situational factors that influence parental feeding decisions when it comes to the use of squeeze pouches such as convenience and cost and for behavioural management.

Across participant accounts, convenience was the most frequently cited driver of pouch use. Parents commonly described squeeze pouches as practical solutions in busy, unpredictable environments—particularly when away from home or short on time. One parent explained they were ‘used for convenience, when travelling/haven’t made something’ (female, aged 33, child aged 6 months–1 year), while others noted their role in school lunchboxes, stating, for example, ‘pouch yoghurt is something my 9 yr takes to school daily’ (female, aged 41, child aged 6–12 years) and ‘5 days a week in their school lunches’ (female, aged 42, child aged 6–12 years).

Cost was another recurring behavioural influence, often discouraging parents from purchase and use. Several parents described tension between the convenience of pouches and their perceived expense. While one parent commented, ‘It’s great when the yoghurts are on special otherwise they are too expensive’ (female, aged 42, child aged 6–12 years), others expressed stronger aversions: ‘Refuse to purchase these expensive products’ (female, aged 54, child aged 6–12 years). Despite this, some parents acknowledged still using pouches in limited circumstances, often when provided by others or heavily discounted: ‘Only were eaten when given away by a company for a promotion and another time when a brand was reduced for quick sale’ (male, aged 51, child aged 6–12 years).

Parents also described pouches as a tool for mess management. Compared to other food options, they were viewed as a cleaner, more contained feeding method. One parent described them as ‘less messy than other options’ (female, aged 35, child aged 2–5 years) while another called them a ‘mess-free alternative’ (female, aged 33, child aged 2–5 years).

A deeper tension emerged in what we refer to as a ‘societal paradox’, wherein the time and financial constraints of modern society prevent parents from being able to make food choices that align with their personal goals and values related to mitigating the nutritional and environmental challenges of our time. Some parents openly criticized the environmental impact of pouches—*‘*an environmental disaster’—yet admitted to still using them on occasion, such as ‘when we are at the supermarket’ or ‘for travel days’ (female, aged 44, child aged 6–12 years). These comments highlighted a gap between feeding ideals and real-world decisions. One mother described this internal conflict clearly: ‘I feel a huge amount of guilt that my 10-month-old eats from pouches, but as a busy single mum I’m struggling to see how to cut them out entirely. They are a huge help’ (female, aged 21, child aged 6 months–1 year).

Squeeze pouches were commonly used to manage children’s behaviour, including as ‘treats’, ‘rewards’, or tools to de-escalate situations. One parent shared, ‘My kids barely eat sweets, so we use fruit pouches instead of lollies’ (female, 41, children 1–2 and 2–5 years). Others used them as incentives: ‘They’re not part of our usual snacks, but I do use them as a reward if we’re shopping and she’s behaving’ (female, 29, child 2–5 years), or for calming: ‘Used as a treat or in emergencies when in public’ (female, 35, child 1–2 years).

Pouches were also a fallback for managing fussy eating. One parent kept one ‘in case of a meltdown’ (female, 34, child 2–5 years), while another said, ‘If nothing is eaten at dinner, I’ll give a pouch so she’s not hungry’ (female, 32, child 1–2 years). Some parents also used pouches as snack options to bridge time between meals or to ensure fullness at bedtime. One mother explained: ‘Used when time is short to keep them tied over until meal times’ (female, aged 31, children aged 1–2 years and 2–5 years), while another noted: ‘We used them when baby was refusing to eat much of her meal but we wanted to make sure she was full before bed’ (female, aged 28, child aged 1–2 years).

For children reported by parents as neurodivergent, pouches were described as safe, predictable foods not just for nutrition, but to support feeding in complex behavioural contexts. One parent noted, ‘He’ll ONLY eat lactose free pouch yoghurt or fruit puree when unregulated’ (female, 41, child 2–5 years). Another shared: ‘My child is on the autism spectrum… she will happily eat fruits and yoghurt from squeeze pouches’ (female, 39, child 2–5 years).

For some parents, the use of pouches by grandparents or external caregivers created dissonance between parental preferences and actual child feeding behaviour.

### Feeding supplement strategies

This theme highlights the parental-reported roles of squeeze pouches in supplementing mealtimes, filling perceived nutrient gaps and managing or maintaining dietary intake during child illness or in periods of selective eating.

Squeeze pouches were often positioned by parents as supplements to main meals, with many using them to boost or fill nutritional gaps. Parents commonly mixed pouches into cereals or used them to fortify meals: ‘From 6 months–18 months my daughter was consuming mainly fruit pouches that were used to top oats or weetbix’ (female, aged 33, child aged 2–5 years) and ‘fruit puree mixed in with weetbix and cows milk of a morning’ (female, aged 30, child aged 1–2 years). Other parents described their use to enhance protein or vegetable intake when children were fussy or eating selectively: ‘As an addition to his family meals if he’s not full or is struggling with the harder textures’ (female, aged 34, children aged 6 months–1 year, 2–5 years).

In some cases, pouches were used creatively in recipes or baking: ‘Fruit pouches in baking recipes’ (female, aged 31, child aged 2–5 years). For others, they were perceived as helpful during illness, teething, or recovery periods: ‘Sometimes when he is sick and won’t eat normal solid foods and the pouches are the only thing he’ll eat’ (female, aged 33, child aged 1–2 years) and ‘When she’s sick or teething and her food consumption drops, I know she’ll have the pouches’ (female, aged 31, child 1–2 years).

Using squeeze pouches to introduce dietary variety was also mentioned, particularly during early feeding. One parent reflected: ‘We used some [squeeze pouches] of fruit and vegetables when first introducing solids’ (female, aged 37, children aged 1–2 years and 2–5 years). Others described the products as providing functional health benefits, especially for digestion: ‘They help add more fibre so that he doesn’t get constipated’ (female, aged 27, child aged 2–5 years).

### Feeding confidence and nutritional perceptions

This theme reflects parents’ perceptions of how squeeze pouches influence children's nutrition and feeding-relating development, including their knowledge and skills for self-feeding, exposure to diverse food textures and overall dietary progression.

Several parents described relying on squeeze pouches as a safe and reliable food option due to limited confidence in preparing meals or knowing what to feed their children. One father shared: ‘We give the kid one of these every so often so that she has something to eat during our mealtimes. We give it because we don't know what to cook for the kid’ (male, aged 41, child aged 6 months–1 year).

Parental perceptions of nutritional value varied considerably. While some viewed them as a healthy option, such as to ‘Provide my toddler with key nutrients’ (female, 35, child aged 2–5 years). Others were more cautious, referring to pouches as ‘sort of nutritious’ (female, aged 38, children aged 1–2 years, 2–5 years and 6–12 years), suggesting uncertainty or awareness of compromise when using them. Many parents considered pouches to be superior in nutritional value compared to shelf-stable options: ‘I feel like this [yoghurt pouch] is a healthy food compared to other packaged snacks which is why I buy them’ (female, aged 37, children aged 2–5 years and 6–12 years).

Autonomous feeding was also a recurring justification for squeeze pouch use. Some parents described how pouches enabled children to self-feed without adult assistance, particularly in structured environments like preschool: ‘I send yoghurt pouches to preschool to foster independence – able to open without teacher assistance’ (demographic data missing, children aged 0–6 months and 2–5 years). Others noted how their child ‘likes them because it’s easy to self-feed’ (female, aged 34, child aged 1–2 years) and that the minimal effort required could increase overall intake: ‘Children are eating more when fed from a pouch because of the minimal effort involved’ (female, aged 21, child aged 6 months–1 year). Yet, even among parents aware of the developmental trade-offs, many felt the benefits of convenience justified their use: ‘I realise they don’t teach my kids great feeding skills… [but] sometimes a Mum of 2 kids needs something easy to offer that’s healthy’ (demographic data missing, children aged 1–2 years and 2–5 years).

### Commercial food environment

This theme describes the role of commercial determinants such as the marketing, labelling, branding, pricing, and availability of squeeze pouches in shaping parental purchasing and usage behaviours.

Parents expressed varying degrees of awareness regarding the marketing and packaging of squeeze pouches. Some, particularly older parents (46+ years of age), were highly conscious of ultra-processed foods and avoided them for this reason, with one participant stating: ‘I would never give those ultra processed foods to my child’ (female, aged 53, child aged 13–17 years). Others criticized the excessive packaging and marketing tactics: ‘Ridiculous packaging, and big food marketing con’ (female, aged 61, child aged 13–17 years).

Despite this, price and promotional strategies often shaped purchasing habits. Many parents reported only buying pouches ‘when on special at the supermarket.’ (female, aged 42, child aged 2–5 years). Marketing claims about ingredients or perceived healthiness also influenced decisions. One parent said: ‘I always aim to buy ‘kids’ and look at the ingredients for no added sugar’ (female, aged 33, children aged 2–5 years) while others shared they prefer to purchase adult-style pouches instead of those marketed towards children: ‘I prefer to buy ‘adult’ style yoghurts in pouch form rather than those promoted for children, but the portions are usually too large for a child’ (female, aged 39, children aged 2–5 years and 6–12 years).

Brand preference was another factor, with many parents favouring certain companies perceived as more ‘natural’ or less processed: ‘We’re very picky with brands’ (female, aged 36, children aged 0–6 months and 2–5 years). One mother said, with others choosing brands with ‘fewer ingredients’ or ‘no added sugar’ (female, aged 35, children aged 2–5 years and 6–12 years).

### Environmental impact considerations

This theme highlights the parental perceptions of single-use packaging waste, strategies for reuse, and tensions between sustainability values and the convenience of squeeze pouches.

Parents expressed strong concerns about the sustainability of commercial squeeze pouch packaging. Several noted they avoided these products entirely due to their environmental impact: ‘They’re not environmentally friendly’ (female, aged 36, child aged 2–5 years). However, others attempted to navigate these concerns by using reusable pouches, often filled with yoghurt. One parent noted: ‘We fill reusable pouches ourselves. It’s more cost-effective’ (female, 34 years, children aged 1–2 years, 2–5 years, 6–12 years, and 13–17 years).

## DISCUSSION

This study provides new insight into how Australian parents and caregivers navigate the use of commercial squeeze pouch products in infant and child feeding. Although the thematic analysis was inductive, the findings point to the significant influence of social and commercial determinants of health on feeding practices. While individual preferences and beliefs were evident, many decisions appeared to be shaped by structural factors, including time constraints, cost, marketing practices, and the broader food environment. Notably, some parents were motivated to reduce waste, yet these accounts suggest there may be limited awareness of how the method of food delivery can affect feeding development. These findings align with international evidence suggesting that the use of packaged baby foods is not solely a matter of individual choice but is embedded within systemic conditions that shape and constrain parental agency (Isaacs *et al*. 2022, Hollinrake *et al*. 2024).

In our study, parental reliance on commercial squeeze pouches appeared to be strongly shaped by commercial determinants, particularly the marketing, availability, and design of these products. While some participants expressed scepticism about marketing and ultra-processed content, most parents described pouches as convenient, portable, and reliable, perceiving them as healthy, with many citing packaging claims such as ‘no added sugar’ and ‘mess free’ when explaining their decisions to use them. This aligns with previous work showing how promotional language and health claims on toddler foods can create a ‘health halo’ effect, confusing parents, and encouraging use even when products are high in free sugars or lack texture diversity (García *et al*. 2019, Dunford *et al*. 2024). The increasing availability, coupled with misleading labelling and front-of-pack claims made by manufacturers, makes these products an appealing choice for families seeking convenient options to feed their children. In some cases, commercial branding and affordability influenced parental trust and purchasing behaviours, particularly among parents with limited confidence and knowledge—reflecting wider research highlighting that parents often receive conflicting advice about infant feeding, which may in turn undermine confidence and decision-making (Harrison *et al*. 2017, Matvienko-Sikar *et al*. 2018). While in other cases, parents compared child-targeted pouches with ‘adult’ options which they perceived the be healthier. These findings reinforce international concerns about the light regulatory environment governing infant and toddler food marketing (Reeve 2016), the conflicting advice offered to parents (Matvienko-Sikar *et al*. 2018), and the expansion of commercial complementary foods into older childhood age groups (da Rocha *et al*. 2021). Current FSANZ reform efforts may provide a timely policy lever to address these gaps.

Beyond marketing, social and structural pressures played a central role in feeding decisions. Parents described the use of pouches to manage work schedules, commuting, and care routines which aligns with broader literature on the influence of time scarcity on food practices (Patrick and Nicklas 2005) and reflects changing social norms around the places and spaces with which children consume food (Higgs 2015). The frequent use of these products is underpinned by convenience, especially when outside of the home, which is also recognized in wider research as a common driver of use (Maslin *et al*. 2015, Isaacs *et al*. 2022, Rowan *et al*. 2022). Additionally, parents described cost-related tensions, with some noting price sensitivity, while others viewed pouches as cost-effective relative to food waste or time and effort associated with food preparation at home. The reassurance parents are offered by marketing claims, combined with the frequent discounts and advertising by grocery stores, is concerning, especially for low-income families or those experiencing food insecurity as these products may displace core foods (Spyreli *et al*. 2021, Isaacs *et al*. 2022, Neve *et al*. 2024).

The accounts in our study reflect tensions between structural constraints and food-related values more broadly, with cost and convenience sometimes overriding nutritional considerations (Baxter *et al*. 2024). Extending this, many participants described using pouches to navigate complex feeding contexts, including managing fussy eating. Similar to our findings, a study in the UK reported that parents frequently used processed baby snacks for non-nutritive purposes such as calming or distracting infants and were influenced by on-pack marketing (Rhodes *et al*. 2025). A recent study also found that some parents choose squeeze pouches over typical children’s menu items, believing they are healthier than discretionary foods (Neve *et al*. 2024), a theme echoed in our findings. Further, the UK survey identified many parents introduced these products earlier than recommended, reflecting how commercial cues can override public health guidance (Rhodes *et al*. 2025). Additionally, parents of children with neurodivergent conditions in our study described pouches as predictable and stress-reducing. While these products may reduce caregiver stress, they may also limit children’s exposure to textures and family foods, raising concerns about feeding development and longer-term health and nutrition (Mathew *et al*. 2022). Evidence suggests extended reliance on commercial squeeze pouches may be associated with delayed texture progression and lower dietary variety (Brunacci *et al*. 2023).

A recurring tension emerged between parental feeding ideals and their actual practices, a paradox also documented in other infant and child feeding research (Petrunoff *et al*. 2014). Parents expressed either ambivalence or guilt about using pouches, describing them as environmentally unsustainable or nutritionally suboptimal, but necessary under various circumstances. Some parents distanced themselves from their use, framing pouch feeding as inconsistent with their usual practices despite resorting to them for convenience. This reflected a societal paradox between parents’ need for convenience due to time and cost factors and guilt over using processed foods in environmentally detrimental packaging, a sentiment echoed in another study of Australian mothers (Begley *et al*. 2019). Others noted that external caregivers used pouches despite family preferences to limit them. This further illustrates the limits of individual-level responsibility framing around nutrition, such that even parents with high food literacy and awareness of nutritional guidelines, and a desire to follow them, were constrained by daily realities. Research from New Zealand has shown higher pouch use in households experiencing severe food insecurity, indicating that structural vulnerability, rather than awareness alone, may be a key driver of commercial infant, toddler and child food consumption (Katiforis *et al*. 2024).

These findings suggest several potential directions for public health and policy. First, there may be value in reviewing the composition and marketing of commercial infant and toddler food products to ensure better alignment with public health guidance. This could include clearer information about appropriate age of use, sugar content, and front-of-pack labelling as well as legislation covering the marketing and labelling of infant foods, labelling of pouches marketed to older age groups, and guidance on the use of pouches and alternative feeding options (Koletzko *et al*. 2019, Moding *et al*. 2019, Katiforis *et al*. 2021, Brunacci *et al*. 2023). Second, there is a need to consider how feeding support for parents can more effectively reflect the realities of everyday caregiving. This might include practical resources delivered through existing child and family health services, as well as greater attention to affordability and cultural relevance of food options. Given that food insecurity has been associated with higher use of squeeze pouches (Katiforis *et al*. 2024), the provision of squeeze pouches by food relief should be carefully considered so as to ensure that families reliant on food relief have a variety of food options for their children. Studies have shown that parents concerned about their cooking skills and ability to prepare meals often use squeeze pouches, believing them to be healthy generally or relative to other options (Isaacs *et al*. 2022, Neve *et al*. 2024), which was a perspective also highlighted in our study. This presents a potential avenue for future public health programmes aimed at ensuring that parents are equipped with the knowledge and skills to prepare simple, age-appropriate, and nutritious meals for their children. Finally, public health messaging and interventions may be more effective if they take into account and address the broader social and commercial contexts in which feeding decisions are made, rather than relying solely on individual education or behaviour change approaches.

### Strengths and limitations

This study provides novel Australian data on the reasons underpinning growing squeeze pouch use, drawing on a diverse sample of parents across age groups and household contexts. However, limitations should be noted. The sample was not representative of the general Australian parental population, with a high proportion of female and university-educated participants compared to national figures (51% female; 63% of people aged 15–74 years hold a non-school qualification) (Australian Institute of Health and Welfare 2023, Australian Bureau of Statistics 2024). This may underestimate challenges encountered by families with lived experience of disadvantage, such as those experiencing food insecurity, lower incomes and education levels, and limit generalizability to other settings. Also, data on the context of food delivery, such as whether contents were consumed directly from the nozzle, were not collected. While allowing for a large sample and amount of qualitative data, the open-text survey format limited opportunities for clarification or probing. As responses were derived from a single open-ended question, it was not always possible to determine which product type parents were referring to, therefore, only responses that explicitly mentioned squeeze pouches were included in the qualitative analysis. Ambiguous quotes were excluded to avoid misinterpretation, with the researchers acknowledging that some relevant insights may have been omitted as a result. Therefore, future qualitative work should explore feeding practices among culturally diverse and lower-income families, and within specific health contexts such as neurodevelopmental diagnoses, and should consider the context in which commercial squeeze pouches are delivered and consumed to better understand their impact on feeding development. Finally, while the findings of this study complement the emerging international literature about squeeze pouch marketing, content, and use, qualitative research in different contexts is needed to elucidate the context-specific drivers of use and opportunities for public health nutrition interventions.

## CONCLUSION

The market for commercial squeeze pouches has rapidly grown over the past decade. This study illustrates that parents’ decisions to purchase and use them to feed their children are shaped by a combination of social and commercial determinants of health. Convenience, cost, time scarcity, and child-specific feeding challenges interact with food marketing, labelling, and packaging claims to create a food environment that supports routine consumption of commercial squeeze pouches. Public health responses could therefore include not only individual education to support parents in making nutritionally and developmentally appropriate food choices but also regulatory, structural, and service-level strategies such as clearer labelling, marketing restrictions, and changes to their nutritional composition to enable parents to feed their children in ways that are nutritionally adequate, developmentally supportive, and environmentally sustainable.

## Data Availability

The data underlying this article will be shared on reasonable request to the corresponding author.
